# Prognostic Value of Metastatic N1 Lymph Node Ratio and Angiolymphatic Invasion in Patients With Pathologic Stage IIA Non-Small Cell Lung Cancer

**DOI:** 10.1097/MD.0000000000000102

**Published:** 2014-10-24

**Authors:** Ching-Feng Wu, Ching-Yang Wu, Jui-Ying Fu, Chi-Wei Wang, Yun-Hen Liu, Ming-Ju Hsieh, Yi-Cheng Wu

**Affiliations:** Division of Thoracic and Cardiovascular Surgery (C-FW, C-YW, Y-HL, M-JH, Y-CW), Department of Surgery; Division of Pulmonary and Critical Care (J-YF), Department of Internal Medicine; and Division of Pathology (C-WW), Chang Gung Memorial Hospital, Chang Gung University, Taoyuan, Taiwan.

## Abstract

With regard to pathologic stage IIA (pIIA) non-small cell lung cancer (NSCLC), there is a paucity of literature evaluating the risk factors for disease-free survival (DFS) and overall survival (OS). The aim of this study was to identify the prognostic factors of DFS and OS in patients with NSCLC pIIA.

We performed a retrospective review of 98 stage II patients (7th edition of the American Joint Committee on Cancer) who underwent lung resection from January 2005 to February 2011. Of these, 23 patients were excluded for this study because of loss of follow-up or different substage, and 75 patients with pIIA were included for further univariate and multivariate analysis. Risk factors for DFS and OS were analyzed, including age, gender, smoking history, operation method, histology, differential grade, visceral pleural invasion, angiolymphatic invasion, and metastatic N1 lymph node ratio (LNR).

Of the 75 patients with pIIA NSCLC who were examined, 29 were female and 46 were male, with a mean age of 61.8 years (range: 34–83 years). The average tumor size was 3.188 cm (range: 1.10–6.0 cm). Under univariate analysis, angiolymphatic invasion and metastatic N1 LNR were risk factors for DFS (*P* = 0.011, *P* = 0.007). Under multivariate analysis, angiolymphatic invasion and metastatic N1 LNR were all independent risk factors for DFS, while adjuvant chemotherapy and higher metastatic N1 LNR were independent prognostic factors for OS.

For patients with pIIA, higher metastatic N1 LNR and angiolymphatic invasion were related to poor DFS. In addition to DFS, higher metastatic N1 LNR was also a poor prognostic factor for OS rates and adjuvant therapy effectiveness. Clinical physicians should devise different postsurgical follow-up programs depending on these factors, especially for patients with high risk.

## INTRODUCTION

Lung cancer is the leading cause of cancer-related mortality worldwide.^1^ Surgical resection constitutes the primary therapeutic option for the management of early-stage non-small cell lung cancer (NSCLC).^2^ According to the 7th edition of the American Joint Committee on Cancer (AJCC), the present pathologic stage II (pIIA) disease has been divided into 6 subgroups, which include T1aN1M0, T1bN1M0, T2aN1M0,T2bN0M0, T2bN1M0, and T3N0M0.^1^ In the 7th edition, previous stage IB subgroup T2bN0 was downgraded to stage IIA and pevious stage IIB subgroup T2aN1 was upgraded to stage IIA. Stage migration indeed happened in the new classification. A review of the literature reveals that Wang et al^3^ found different 5-year overall survival (OS) between pIIA and pIIB (59.7% vs. 47.2%).^3^ This means that pIIA and pIIB were different groups of patients and should be discussed separately. The special characteristic of stage II is N1 lymph node (LN) involvement. During the past 2 decades, many studies have evaluated the validity of the N descriptors and have suggested refinements that would allow more accurate prognostic stratification.^4,5^ These findings have generated considerable interest in identifying N1 LN involvement in order to accurately predict survival.^6^ Patients who are N1-positive suffer a considerable risk of recurrence; therefore, such patients may need aggressive postoperative therapy.^7–10^ Recently, many authors have suggested that within the subset of patients with pN1 disease, prognosis may differ based on the metastatic lymphatic ratio.^11,12^ Several studies have suggested that the ratio of involved to non-involved nodes may be an alternative, and possibly better, indicator of tumor burden and consequently disease prognosis than pN staging. The aim of this study was to evaluate surgical–pathological factors that affect prognosis of patients having pIIA NSCLC with particular emphasis on the prognostic significance of N1 lymph node ratio (LNR) at a single institution.

## MATERIAL AND METHODS

### Patients

We performed a retrospective review of 98 stage II patients (7th edition of AJCC^1^) who underwent lung resection from January 2005 to February 2011. Exclusion criteria included an incomplete medical record and patients becoming lost to follow-up or patients whose adjuvant chemotherapy was discontinued because of adverse events. Twenty-three patients were excluded for this study because of the loss to follow-up or different substage (5 patients were lost to follow-up, 6 patients were T3N0, and 12 patients were T2bN1). Among the 75 enrolled patients, 56 received cisplatin-based chemotherapy after their operation, 10 received complete 2-year uracil–tegafur (UFT) treatments, and 9 did not receive any adjuvant chemotherapy after their operation. The preoperative workup included chest radiography, bronchoscopy, chest computed tomography (CT), spirometry, bone scan, and a thorough search for distant metastases, including positron emission tomography (PET) imaging. The study was approved by the Institutional Review Board (IRB), and the IRB number was 99-1586B.

### Surgical Technique

Lobectomy, bilobectomy, or pneumonectomy with systemic lymphadenectomy were performed according to our institutional policy. All pulmonary resections were performed by open thoracotomy (Open) or video-assisted thoracoscopic surgery (VATS). Surgical resections included 2 pneumonectomies, 3 bilobectomies, and 70 lobectomies.

Complete anatomic resection was achieved in all the patients. Although the number of intrapulmonary and mediastinal LNs is highly variable from one patient to another, with no relevant impact on OS,^13^ we did complete hilar and mediastinal lymphadenectomy for every case.

### Postoperative Adjuvant Therapy

For 10 patients classified as pIB with 6th AJCC, UFT was given as a postoperative adjuvant therapy, starting from the 4th week postoperative and continued for 2 years.^14^ Most patients received 2 capsules of UFT (200 mg of tegafur and 448 mg of uracil), twice daily. The dose was rounded to the nearest 100 mg. At each follow-up visit, treatment compliance and drug-related adverse events were evaluated. For the 56 patients classified as pIIA with 6th AJCC, 4 or 6 cycles of cisplatin-based chemotherapy regimen were given as postoperative adjuvant therapy; blood count (white blood cells >3.0 × 10^3^; absolute neutrophil count >1500; platelets >100 × 10^3^), liver function (aspartate aminotransferase/alanine aminotransferase and alkaline phosphatase <2 times the upper limit of normal was accepted), and renal function (serum creatinine ≤2 mg/dL via biochemistry tests) were monitored in all the patients prior to adjuvant chemotherapy.

### Pathological Evaluation

According to the TMN classification of the 7th AJCC staging, all patients were staged as final pIIA. The pathologically recorded variables included tumor size, tumor differential grade, visceral pleural invasion,^15,16^ angiolymphatic invasion,^17–19^ tumor histology, and metastatic N1 LNR.^6^

### Follow-Up

The patients were examined on an outpatient basis at 3-month intervals for the first 5 years and at 6-month intervals thereafter. Follow-up evaluation included physical examination, chest radiography or chest CT, brain magnetic resonance imaging, and ^18^F-fluorodeoxyglucose PET. Recurrent NSCLC was diagnosed on the basis of physical examination and diagnostic imaging of lesions consistent with recurrent lung cancer. Follow-up examinations were continued until February 28, 2014.

### Statistical Analysis

Statistical analysis was done with SPSS (V17.0, SPSS, Inc, Chicago, IL). Categorical variables were compared using the χ^2^ test, while continuous variables were compared with the *t* test. OS was defined as the time from surgery to death or to the last follow-up visit. OS curves were estimated using the Kaplan–Meier method. Significance was assessed using the log rank test. A *P* value of <0.05 was considered to indicate statistical significance.

## RESULTS

Of the 75 patients with pIIA NSCLC who were examined, 29 were female and 46 were male, with a mean age of 61.8 years (range: 34–83 years). The average tumor size was 3.188 cm (range: 1.10–6.0 cm). Angiolymphatic invasion was seen in 38 patients (50.7%) and visceral pleural invasion was noted in 29 patients (38.7%). The mean survival time was 5.514 years (range: 0.18–8.82 years), and the median survival time was 5.91 years. The characteristics of patients’ profiles are shown in Table 1.

**TABLE 1:**
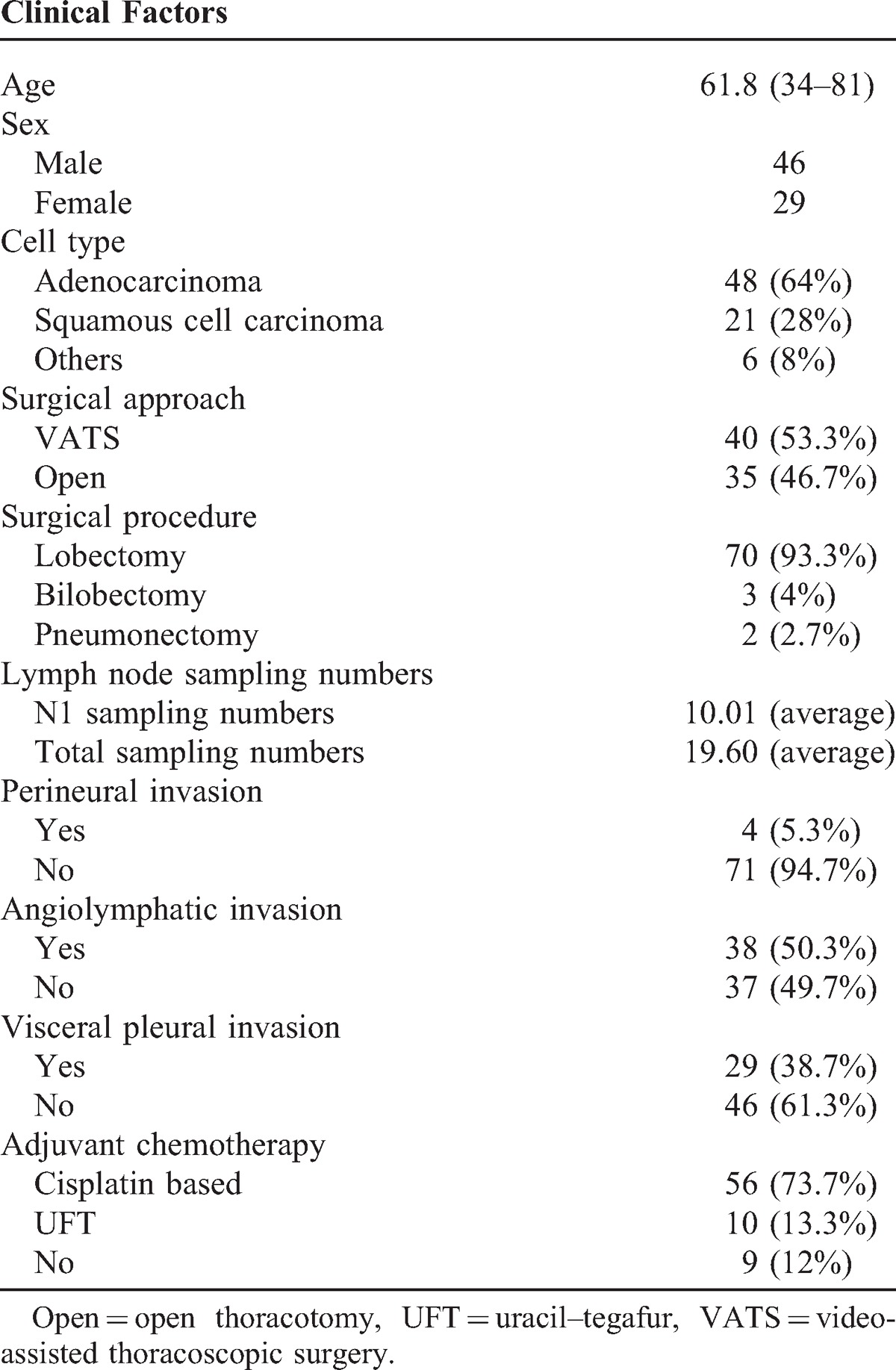
Patient Demographics and Characteristics

**FIGURE 1:**
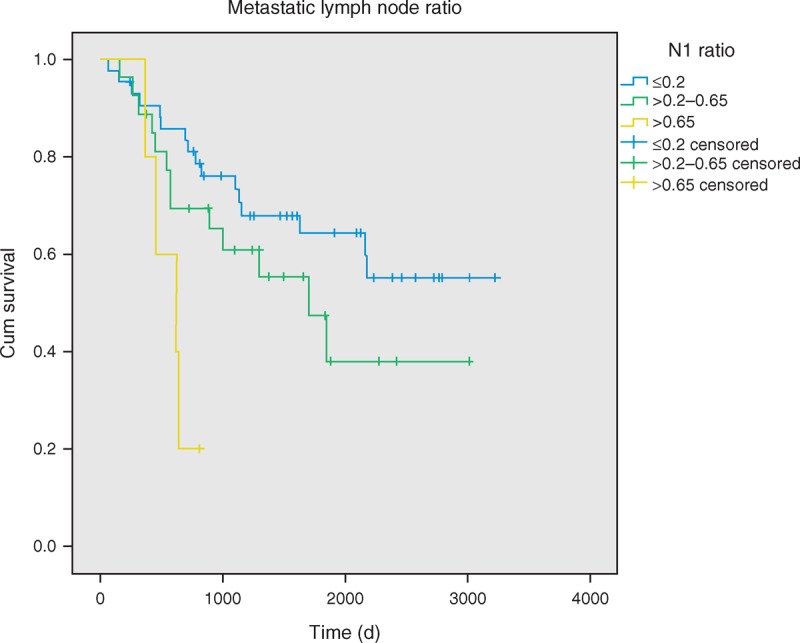
Overall survival of pathologic stage IIA patients with metastatic lymph node ratio, *P* = 0.011.

For all the patients, the 5-year survival rate after surgery was 55%. Smokers had a worse prognosis in OS (*P* = 0.015). The 5-year survival rates for adenocarcinoma and nonadenocarcinoma patients were 54% and 50%, respectively, showing no statistical difference (*P* = 0.299). Adjuvant therapy seemed to prolong the patients’ OS (*P* = 0.015). Metastatic N1 LNR was classified into 3 groups, including LNR ≤ 0.2, 0.2 < LNR ≤ 0.65, and LNR > 0.65. We found that patients with lower metastatic LNR had significantly better survival rates than those with higher metastatic LNR, with 5-year survival rates of 64%, 45%, and 20%, respectively (*P* = 0.011; Figure 1). For the 66 patients who received adjuvant therapy, lower metastatic LNR had a better survival curve than higher metastatic LNR (*P* = 0.004). No difference in OS was observed with regard to gender and age, visceral pleural invasion, tumor differentiation grade, tumor size, angiolymphatic invasion, or types of operation method (VATS vs. Open).

In all stage IIA cases, median disease-free survival (DFS) lasted 3.70 years, and 1-year, 3-year, and 5-year DFS rates were 70%, 44%, and 34%, respectively. The 5-year DFS rates of patients with and without angiolymphatic invasion were 16% and 46%, respectively (*P* = 0.011). DFS was shown to be significantly longer in patients with lower metastatic N1 LNR. These patients had an average 5-year DFS rate of 50%, as opposed to 22% and 20% (*P* = 0.007). No difference in DFS was detected with regard to patient’s gender, smokers or nonsmokers, age, visceral pleural invasion, tumor differentiation grade, and tumor size.

**FIGURE 2:**
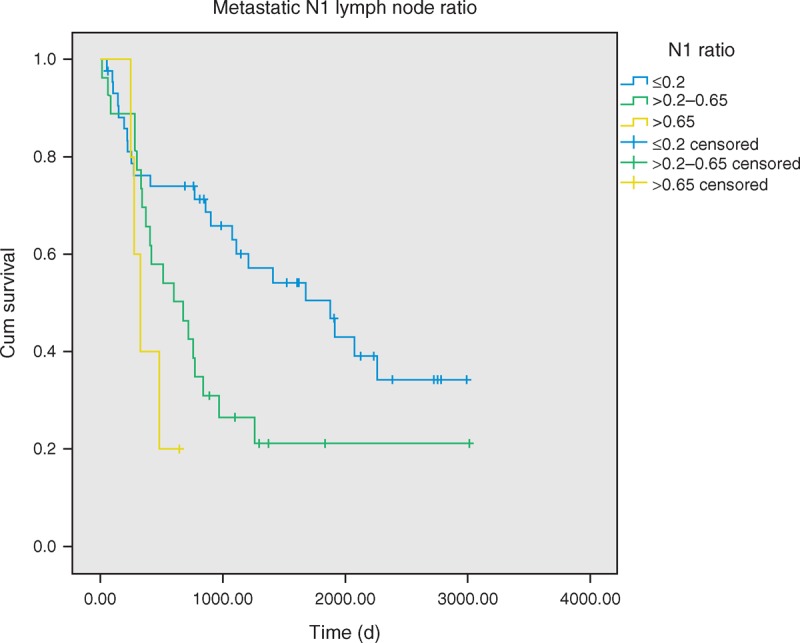
Disease-free survival of pathologic stage IIA patients with metastatic lymph node ratio, *P* = 0.008.

**FIGURE 3:**
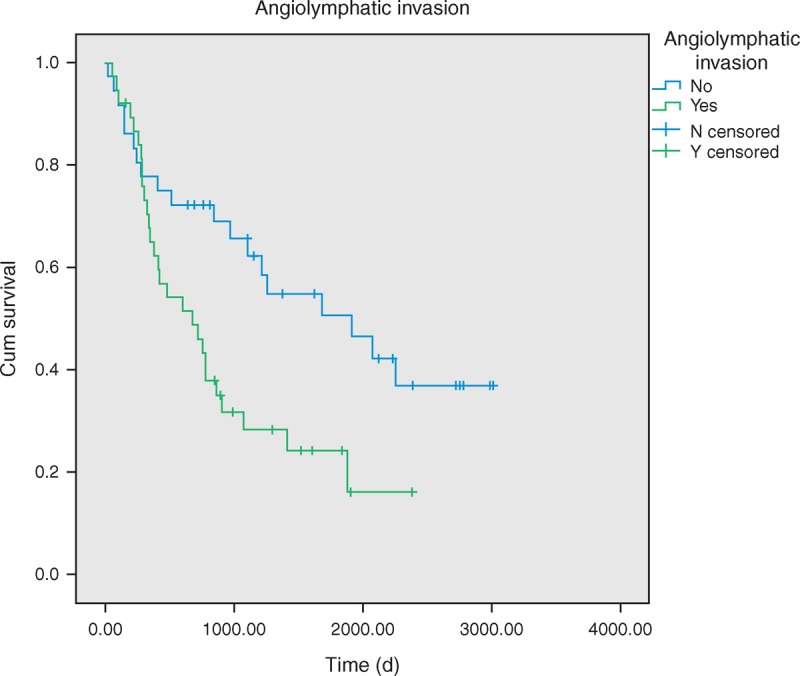
Disease-free survival of pathologic stage IIA patients with/without angiolymphatic invasion, *P* = 0.011.

The univariate analyses indicated that the significant factors, smoking habit and higher LNR, were associated with OS (Table 2). Patients with angiolymphatic invasion (*P* = 0.011) and higher LNR (*P* = 0.011) have worse DFS rates (Figures 2 and 3). In the multivariate analysis, possible prognostic factors associated with DFS and OS were considered in a multivariable Cox proportional hazard regression analysis and are presented in Table 3. Metastatic N1 LNR was the risk factor for DFS and OS. Angiolymphatic invasion was associated with poor DFS (hazard ratio: 1.9, 95% confidence interval [CI]: 1.01–3.61, *P* = 0.045). In addition, adjuvant chemotherapy was a good prognostic factor for OS (hazard ratio: 0.31, 95% CI: 0.10–0.92, *P* = 0.035).

**TABLE 2:**
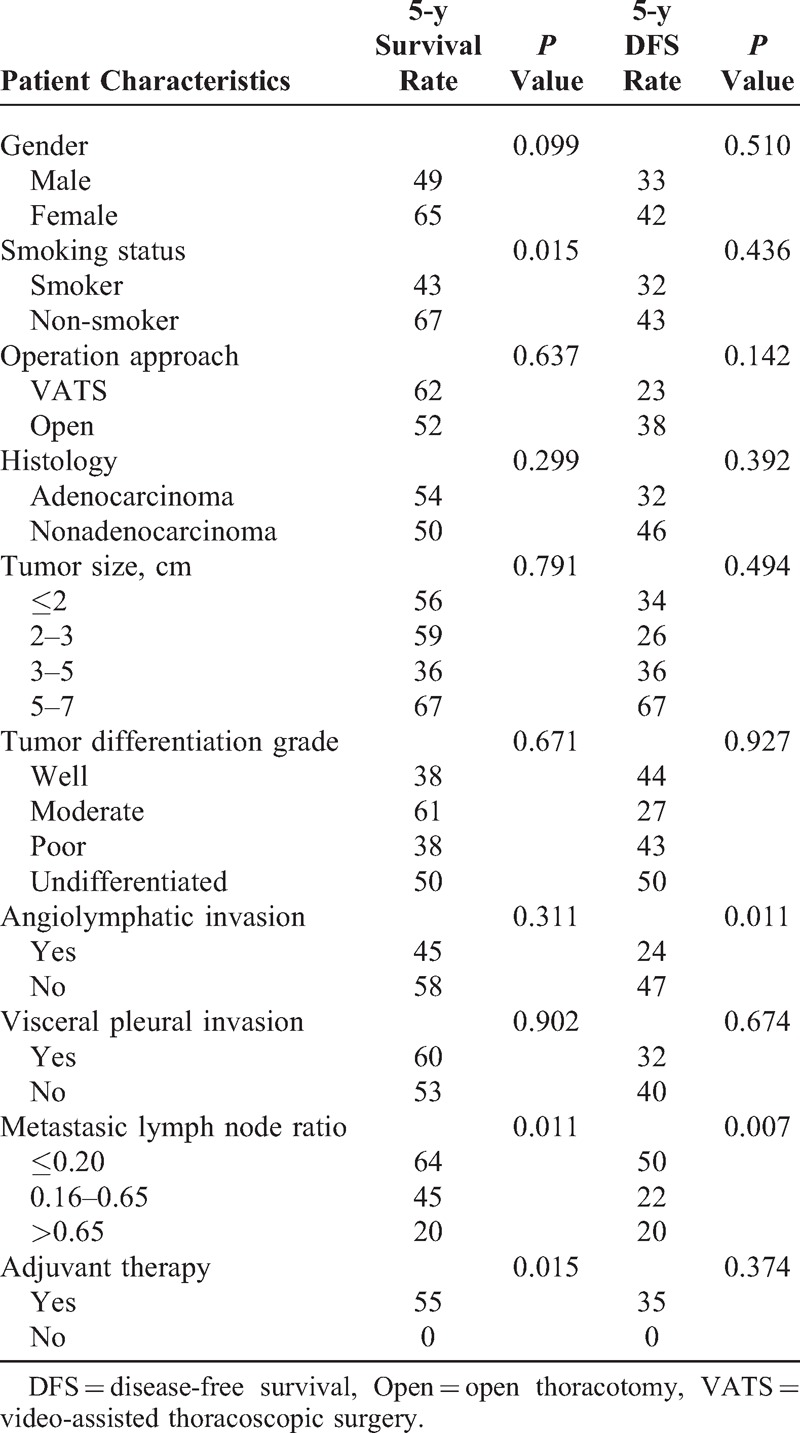
Clinicopathological Risk Factors: Univariate Analysis

**TABLE 3:**
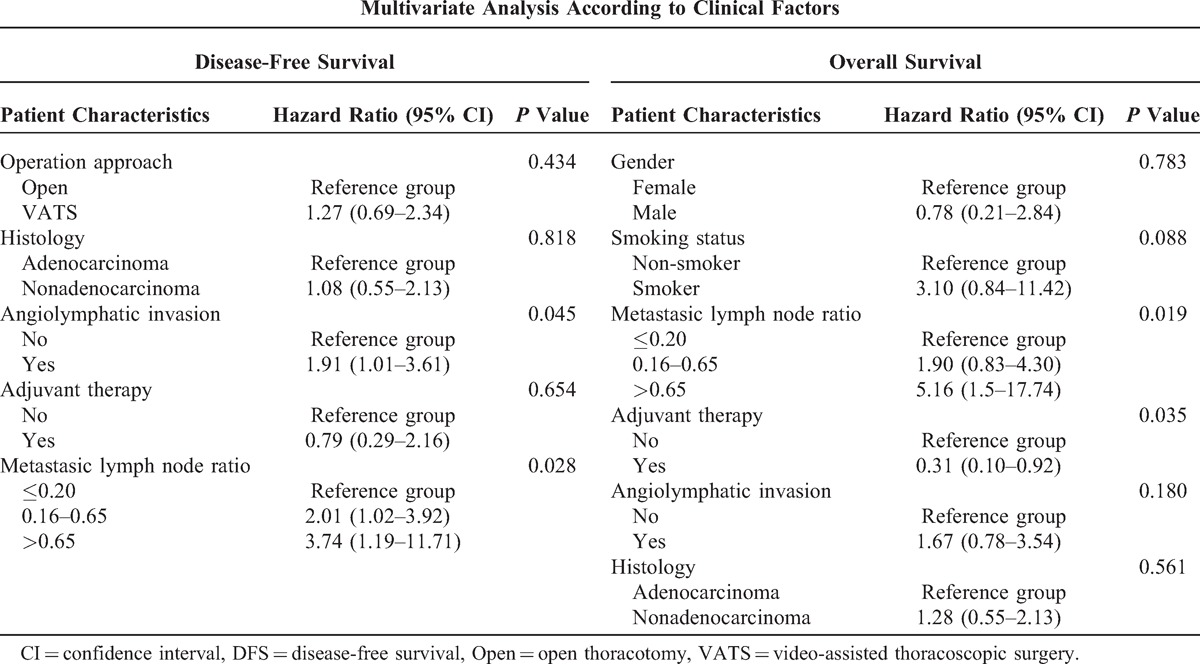
Multivariate Analysis of Overall Survival

## DISCUSSION

According to the International Association for the Study of Lung Cancer Staging Project,^20^ patients with pN1 have worse survival rate than patients with pN0. Although the N descriptors were not changed in the 7th edition of the AJCC, several studies have shown that N1 disease affects a heterogeneous group of patients who have different tumor size or LN-related factors that can affect prognosis.^8^ For clinical practice, a single-stage study is needed for physicians to tailor postoperative management strategies and identify patients who would benefit most from aggressive chemotherapy and follow-up strategies. In our study, 5-year survival rate after surgery was 55%, which is similar to a previous study,^8^ and postoperative adjuvant chemotherapy brought a better survival rate (*P* = 0.015).

From literature review, there is increasing evidence that the involved LNs ratio, that is, number of positive LNs over total resected LNs, may be related to prognosis in many forms of cancer including esophageal, thyroid, breast, periampullary, gastric, colorectal, and cervical cancers.^21–27^ Wisnivesky et al^28^ reported the prognostic impact of the LNR on pN1 NSCLC. They divided their patients into 3 subgroups according to the LNR (<0.15, 0.15–0.5, and >0.5) and found that the OS became significantly worse as the LNR increased. In our study, we identified that patients having stage IIA cancer with lower LNR had longer overall and DFS rates than patients with higher LNR values (*P* = 0.008 and 0.011, respectively). Multivariate analysis found that LNR values were independent predictors of OS and DFS rates (*P* = 0.019, *P* = 0.028). The ratio of involved LNs has also been recognized as a significant predictor of survival in N1-NSCLC. Therefore, the clinical implications of the number of LN involved in N1 disease need further investigation.

In a previous study,^29,30^ angiolymphatic invasion has been shown to be a poor prognostic factor for recurrence-free survival and OS in NSCLC. Higgins et al^31^ showed that angiolymphatic invasion is associated with higher risk of distant metastases and shorter long-term survival in a population of patients with predominantly stage IA and IB tumors. In our study, angiolymphatic invasion was identified as an independent risk factor for DFS but not OS. The relapse pattern, that is, distant or locoregional relapse, was not related to angiolymphatic invasion. This was probably on account of some complex lymphatic lung drainage flow. Some lymph was directly drained to the mediastinum but not to the hilar LN stations.^32^ However, few reports have mentioned its role in stage IIA NSCLC. Thus, further investigation is needed to understand the relationship between angiolymphatic invasion and relapse pattern.

Our study has some limitations. Despite being a retrospective analysis with a limited number of cases, we still were able to identify the risk factors that affect prognosis. Higher metastatic LNR showed poor DFS and OS rates and needs a more aggressive postsurgical follow-up program. Patients with angiolymphatic invasion showed poor DFS rates and, thus, adjuvant therapy is recommended.

## CONCLUSION

For patients with pIIA, higher metastatic N1 LNR and angiolymphatic invasion were related to poor DFS. In addition to DFS, higher metastatic N1 LNR was also a poor prognostic factor for OS rates and adjuvant therapy effectiveness. Clinical physicians should devise different postsurgical follow-up programs depending on these factors, especially for patients with high risk.
